# Correction of Experimental Retinal Ischemia by l-Isomer of Ethylmethylhydroxypyridine Malate

**DOI:** 10.3390/antiox8020034

**Published:** 2019-02-03

**Authors:** Anna Peresypkina, Anton Pazhinsky, Mikhail Pokrovskii, Evgenya Beskhmelnitsyna, Anna Pobeda, Mikhail Korokin

**Affiliations:** Department of Pharmacology and Clinical Pharmacology, Institute of medicine, Belgorod State National Research University, Belgorod 308015, Russia; bb_9393@mail.ru (A.P.); mpokrovsky@yandex.ru (M.P.); evgeny_b89@mail.ru (E.B.); pobeda@bsu.edu.ru (A.P.); mkorokin@mail.ru (M.K.)

**Keywords:** retinal ischemia, l-isomer of ethylmethylhydroxypyridine malate, Emoxipine, b/a coefficient, Wistar rats

## Abstract

An important task of pharmacology and ophtalmology is to find specific and highly effective agents for correcting retinal ischemia. The objective of this study is to increase the effectiveness of pharmacological correction of retinal ischemia by using new 3-hydroxypyridine derivative–l-isomer of ethylmethylhydroxypyridine malate. A modification to the retinal ischemia-reperfusion model was used, in which an increase in intraocular pressure is carried out by mechanical pressure (110 mmHg) to the front chamber of the eye for 30 min. The protective effects of l-isomer of ethylmethylhydroxypyridine malate in comparison with Emoxipine as pretreatment, with parabulbar injection, based on the model of retinal ischemia-reperfusion, were estimated by the changes in the ratio of the amplitudes of the a- and b-waves of electroretinography (the b/a coefficient) and ophthalmoscopy. The use of l-isomer of ethylmethylhydroxypyridine malate improves the retinal electrophysiological state after 72 h of reperfusion; in the group of rats treated with l-isomer of ethylmethylhydroxypyridine malate, the coefficient b/a was reliably increased by 9.5%, *p* < 0.05, in comparison with animals treated with Emoxipine, and by 91.7%, *p* < 0.05, in comparison with the group with no treatment. Furthermore, it prevents the development of ischemic changes in the retina observed in ophthalmoscopy to a greater extent than Emoxipine.

## 1. Introduction

Determining how to optimize tissue tolerance to ischemia is a challenge of experimental pharmacology [1,2,3]. Currently, the treatment of ischemic retinal conditions was done by using pharmacological agents such as angioprotectors, fibrinolytics, vasodilators, anticoagulants and others. Due to the instability effects when carrying out therapy with drugs of the specified pharmacological groups, it is necessary to seek out more effective methods to increase resistance to the ischemia of retinal tissue [4,5]. Therefore, it is important to find specific and highly effective pharmacological agents to correct retinal ischemia.

With retinal ischemia, the optic nerve is primarily affected. Ischemic optic neuropathy is usually characterized by sudden development, but in some cases, there is a progressive visual loss within two weeks [6]. In order to make the correct diagnosis, ophthalmoscopy and electroretinography (ERG) are necessary [7,8]. Analysis of the electroretinogram allows one to evaluate the topography of retinal disorders, as well as the reaction of the retina to the pharmacological correction.

Non-arteritic anterior ischemic optic neuropathy is the most common form of ischemic optic neuropathy. The prevalence of the disease is 2.3–10.2 per 100 000 population over 50 years of age. Men are affected 5 times more often than women. Patients usually have diabetes, arterial hypertension, etc. [9]. Atrophy of the inner retinal layers is observed in non-arteritic ischemic optic neuropathy, but a significant part of the nerve fiber layer and the ganglion cell layer is preserved in patients even with no light perception [10].

As of today, there is no well-proven method to treat non-arteritic anterior ischemic optic neuropathy, despite the fact that it is the main disease that affects the optic nerve in elderly people [11].

Emoxipine (Methylethylpiridinol), a 3-hydroxypyridine derivative, is an antihypoxant, antioxidant [12,13] and angioprotector. The antioxidant action is carried out due to the inhibition of lipid peroxidation and activation of the antioxidant system, modification of metabolic receptors and transport functions of cell membranes, stabilization of calcium-ATPase of sarcoplasmic reticulum in conditions of sarcolemma denaturation, which prevents damage to the cell membrane [14]. Emoxipine is used successfully in Russia, mainly in ophthalmology [15,16]. The use of Emoxipine leads to the resorption of intraocular hemorrhages, reduces capillary permeability, and increases resistance to hypoxia and ischemia [17,18].

Parabulbar injection of 1% Emoxipine solution has a more pronounced protective effect in comparison with instillation; the maximum concentration of Emoxipine in the retina with parabulbar injection is observed after 30 min. [19].

The retinoprotective effects of Emoxipine at a dose of 2 mg/kg on retinal ischemia-reperfusion model in rats have been studied previously, so Emoxipine was chosen by us as a comparison drug [20]. The assumption is that a new 3-hydroxypyridine derivative l-isomer of ethylmethylhydroxypyridine malate will exhibit a protective effect on the model of retinal ischemia-reperfusion.

In view of the above, the relevance of the research can be seen in studying the protective effects of the l-isomer of ethylmethylhydroxypyridine malate along with evaluating the ratio of the amplitudes of the a- and b-waves of ERG (the coefficient b/a) and eye fundus image in rats when ischemia-reperfusion is corrected.

The objective of this study is to increase the effectiveness of pharmacological correction of retinal ischemia by using a new 3-hydroxypyridine derivative—l-isomer of ethylmethylhydroxypyridine malate.

## 2. Materials and Methods

### 2.1. Animals

The experiments were approved by the Belgorod State National Research University, Local Ethics Committee, Belgorod (Protocol#15/18). Ethical principles of handling laboratory animals were observed in accordance with the European Convention for the Protection of Vertebrate Animals Used for Experimental and Other Scientific Purposes, CETS No. 123. The animals were housed in an animal facility with a 12-h day/12-h night cycle and provided a standard laboratory diet and water. Experiments were carried out on 40 Wistar rats (4 groups, 10 animals in each group) weighing 250 ± 25 g. For the study, the rats were taken with no external signs of disease, having passed the quarantine regime.

### 2.2. Experimental Design

The following groups were included in the experiment: the first—a control group; the second—a group with the simulated retinal ischemia-reperfusion; the third—a group with correction of the pathology by l-isomer of ethylmethylhydroxypyridine malate; and the fourth—a group with the correction by Emoxipine.

Retinal ischemia-reperfusion injury was simulated under general anesthesia (chloral hydrate, 300 mg/kg of rat body weight, i.p.) by applying mechanical pressure (110 mm Hg) to the anterior eye chamber for 30 min [21,22]. Mechanical pressure on the anterior chamber was carried out by a metal rod with an atraumatic surface placed in the cylinder with piston system with a calibration scale. The system for retinal ischemia modeling was calibrated as followed: IOP was registered by introducing into the anterior chamber 30G needle sensor (Biopac System, Inc., Goleta, CA, USA), TSD104A hardware-software complex MP150 production Biopac System, Inc. (Goleta, CA, USA) with use the computer program AcqKnowledge 4.2. Rendering mechanical pressure on the anterior chamber was carried out as per step calibration system of the cylinder with the registration of the increase of IOP that allowed us to estimate the level of IOP increase at a certain position.

The substance l-isomer of ethylmethylhydroxypyridine malate was synthesized in the All-Russian Scientific Center for Safety of Biologically Active Substances, Kupavna, Russia. l-isomer of ethylmethylhydroxypyridine malate was injected parabulbarly, through the skin into the *corpus adiposum orbitae*, the area of the lower eyelid, as 1% solution, 0.05 mL once daily for 4 days including the first injection 30 min before ischemia-reperfusion modeling. When conducting parabulbar injections rats, were anesthetized (chloral hydrate, 300 mg/kg of rat body weight, intraperitoneal injection).

Emoxipine (solution for injection, 10 mg/mL, Federal State Unitary Enterprise “Moscow Endocrine Plant”) at a dose of 2 mg/kg was injected parabulbarly [19] in the same manner as l-isomer of ethylmethylhydroxypyridine malate.

In the control group, an equivalent volume of water for injections was injected parabulbarly in the same manner as the studied pharmacological agents.

### 2.3. Electroretinography in Rats

Electroretinography in rats was performed according to the method previously published by us [19,21]. To perform ERG, rats were kept in the dark for 30 min, then anesthetized (chloral hydrate, 300 mg/kg of rat body weight, i.p.) [23]. Corneal silver electrode was placed on the cornea that has been soaked by saline solution for better contact, the reference needle electrode EL452 (Biopac System, Inc., Goleta, CA, USA) has been placed subcutaneously in the region of the skull, ground needle electrode EL450 (Biopac System, Inc., Goleta, CA, USA) has been placed subcutaneously in the base of the tail. A stroboscope TSD122B with a flash of white light connected to the stimulator STM200 (Biopac System, Inc., Goleta, CA, USA) was placed behind the animal’s back. Evoked biopotentials were run at a frequency of 1–1000 Hz, amplified, averaged, and presented graphically on the screen using the MP150 data acquisition and analysis system (Biopac Systems, Inc., Goleta, CA, USA). The duration of the flashes was 0.025 sec, intensity was 30000 Volts. ERG registration was carried out in response to a single stimulation. The ERG recording was carried out for 0.5 sec on each rat in the groups. To assess the degree of retinal ischemia, the ratio of the amplitudes of the a- and b-waves of ERG, the b/a coefficient was evaluated. The mean was derived for each group from ten values received and was introduced into the protocol.

### 2.4. Ophthalmoscopy

The rats were anesthetized to perform ophthalmoscopy. To dilate the pupils, Irifrin drops 2.5% were used. The ophthalmoscope was brought closer to the rat’s eye and a beam of light was directed at the eye from a distance of 0.5–2.0 cm to obtain a clear eye fundus image. When the fundus image was unclear, a proper lens was selected by turning the disk of the ophthalmoscope to provide a clear image of the fundus details.

An Ocular Osher MaxField 78D, model OI-78M (Volk Optical Inc, Mentor, OH, USA), was used to magnify and obtain photos of the eye fundus.

### 2.5. Statistical Analysis.

For all data, descriptive statistics were used, and the data were checked for normal distribution. Distribution type was determined by using the criterion of Shapiro-Wilk. In case of normal distribution, the average value (M) and standard error of the mean (m) were calculated. In cases of abnormal distribution, the median (Me) and the quartile range (QR) were calculated. Between-group differences were analyzed by parametric (*t*-Student criterion) or non-parametric (Mann-Whitney test) methods, depending on the type of distribution. Differences were determined at a 0.05 significance level. Statistical analyses were performed using Statistica 10.0 software.

## 3. Results

### 3.1. Results of Evaluation of Electrophysiological Retinal Function

After the pathology modeling, the ERG was recorded. The ERG serves to register the potential of retinal cells in response to light. The assessment of retinal electrical activity was conducted with a- and b-wave amplitude of the electroretinogram: a-wave is a negative wave reflecting the functional activity of photoreceptors, and b-wave is a positive wave reflecting the electrical activity of bipolar and Muller cells with the possible involvement of the horizontal and amacrine cells [21].

An example of electroretinogram of rat in control group is presented in Figure 1.

Inhibition of the a-wave was not observed in all animals with the simulated retinal ischemia-reperfusion. In cases where the amplitude of the a-wave decreased, the inhibition of the b-wave was more significant. As a rule, changes in the b-wave developed before the suppression of a-wave, and they were more pronounced (Figure 2).

During the correction with the studied pharmacological agents, an increase in b-wave was mainly observed (Figure 3).

The influence of l-isomer of ethylmethylhydroxypyridine malate and Emoxipine on the electrophysiological state of the retina, on the a and b wave amplitudes in particular, when correcting retinal ischemia, after 72 h of reperfusion are presented in Table 1.

In each group, the coefficient b/a was calculated, the values of which are presented in Table 2.

The b/a coefficient in the group with ischemia-reperfusion model was 1.2 ± 0.04, which was significantly different from that of the control group, by 52% (*p* < 0.05). In the group with the correction by l-isomer of ethylmethylhydroxypyridine malate, the b/a coefficient was significantly different from that of the group with retinal ischemia, by 91.7% (*p* < 0.05). In the group with the correction by Emoxipine, the b/a coefficient was significantly different from that of the group with retinal ischemia, by 75% (*p* < 0.05). The increase of this indicator confirms the maintenance of electrophysiological retinal function.

### 3.2. Results of Ophthalmoscopy

The eye fundus image in rats from the control group is as follows: optic disc (OD) is round or oval and pale pink. The boundaries of OD are clear. It is located in the plane of the retina. Out of the middle of the OD come the central vessels of the retina. The overall background is pink (Figure 4a). The fundus image in rats with simulated pathology is as follows: OD is edematous, increased, edema extends to the retina. The boundaries of OD are unclear. There are foci of soft exudate, indicating an increase in ischemia. Hemorrhages (Figure 4b).

In the group with correction by l-isomer of ethylmethylhydroxypyridine malate, the following fundus image was observed: OD is round, non-edematous, pale pink, is located in the plane of the retina, the boundaries are clear (Figure 5a). The fundus image in rats with correction by Emoxipine is as follows: OD is edematous, increased. The boundaries of OD are unclear. There are hemorrhages over the OD (Figure 5b).

Thus, the results of evaluation of electrophysiological retinal function and ophthalmoscopy on the retinal ischemia-reperfusion model showed that the most effective was the correction by l-isomer of ethylmethylhydroxypyridine malate at a dose of 2 mg/kg, exceeding the reference drug Emoxipine at a dose of 2 mg/kg.

## 4. Discussion

The search for new ways to achieve retinoprotection for the possible reduction of the damaging effect of ischemia, formed in various systemic diseases (diabetes, arterial hypertension etc.), is an important task of pharmacology and ophthalmology. Recently, a large amount of research has been devoted to the study of various pharmacological agents with antioxidant activity for the correction of retinal ischemic damage and retinopathy that emphasizes the relevance of the problem [24,25,26,27].

3-hydroxypyridine derivatives (Emoxipine, Russian Federal State Unitary Enterprise «Moscow Endocrine Plant», Moscow, Russia; Mexidol, FARMASOFT Research-and-production complex LLC, Moscow, Russia) are used in the treatment of ischemic optic neuropathy. Emoxipine is used for the treatment of subconjunctival hemorrhages and intraocular hemorrhages, angioretinopathy, central and peripheral chorioretinal dystrophy, thrombosis of the central retinal vein and its branches, etc. [28].

The main effect of Emoxipine is direct antiradical, for the implementation of which 3-hydroxypyridine pharmacophore is responsible. Emoxipine, accumulating in the cytoplasmic membrane of cells, increases its fluidity and reduces viscosity, contributing to the penetration of succinate, and thus increasing its bioavailability. Succinate is an intermediate of the Krebs cycle; its advantage is the transformation through FAD, independent of the presence of NADH, which is important in ischemia. Succinic acid supports the work of the electronic transport chain and prevents the development of mitochondrial dysfunction. Moreover, receptors to succinate (SUCNR1 or GPR91) associated with G-protein have been found, through which it implements a number of regulatory functions, e.g., activating apoptosis in acute ischemia, promoting the release of renin, proangiogenic factors in the retina [29]. A decrease in the activity of succinate dehydrogenase is observed in hypoxia and hypoglycemia, which is accompanied by the accumulation of succinate and the effect on specific receptors.

This study revealed the protective effect of a new 3-hydroxypyridine derivative l-isomer of ethylmethylhydroxypyridine malate on the model of retinal ischemia-reperfusion, which is expressed in the normalization of the functional activity of the retina (ERG) and ophthalmoscopic picture in laboratory rats, and exceeded the protective effect of Emoxipine.

The levorotatory isomer of ethylmethylhydroxypyridine malate was selected for the study, based on the following. It is known that the levorotatory isomer of malic acid participates in the Krebs cycle, since the catalysis occurs stereoselectively. Therefore, d-form can even reduce the energy potential, by competing with l-forms for the enzyme. The presence of such a "molecular trap" may increase the toxicity and reduce the therapeutic latitude. There are experimental data that corroborate the advantage of levorotatory malate over racemic malate: l-malate is 1.5 times more effective than its d-isomer in antihypoxic activity on the model of hypobaric hypoxia [30]. K. Rognstad and O. Katz showed that the incubation of kidney tissue in an environment with d-malate leads to insignificant formation of glucose, which indicates the inhibition of the enzyme malate dehydrogenase [31].

Molecular and cellular processes involved in maintaining the integrity of neural network in nerve tissue damage include a number of mechanisms, e.g., synthesis of neurotrophic factors and cytokinesm expression of proteins supporting cell survival (chaperones, antioxidants, apoptosis inhibitors, etc.), activation of proteins involved in DNA repair, etc.

The presence of 3-hydroxypyridine in structure provides a complex of antioxidant and membranotropic effects, as well as the reduction of glutamate excitotoxicity [32]. The detrimental effect of glutamate on retinal ganglion cells has been shown to involve NMDA receptors through exposure of the retina to high glutamate levels. In excitotoxicity, glutamate triggers the rise of intracellular Ca^2+^ levels, followed by the upregulation of neuronal NOS, dysfunction of mitochondria, reactive oxygen species production, endoplasmic reticulum stress, and release of lysosomal enzymes [33,34,35,36,37].

Therefore, in the future, it is planned to study l-isomer of ethylmethylhydroxypyridine malate on the model of NMDA-induced excitotoxicity in retina to confirm the intended biological target action of this substance.

## 5. Conclusions

The use of l-isomer of ethylmethylhydroxypyridine malate improves retinal electrophysiological state after 72 h of reperfusion on the retinal ischemia-reperfusion model. In the group of rats treated with l-isomer of ethylmethylhydroxypyridine malate, the b/a coefficient was reliably increased by 9.5%, *p* < 0.05, in comparison with animals treated with Emoxipine, and by 91.7%, *p* < 0.05, in comparison with the group with no treatment. Furthermore, it prevents the development of ischemic changes in the retina observed in ophthalmoscopy to a greater extent than Emoxipine.

## Figures and Tables

**Figure 1:**
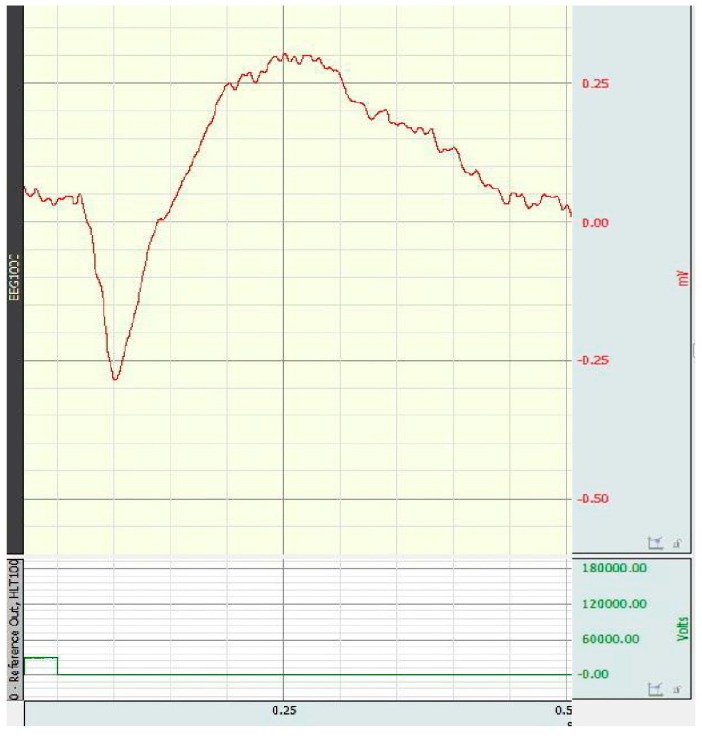
Electroretinogram of rat in control group.

**Figure 2:**
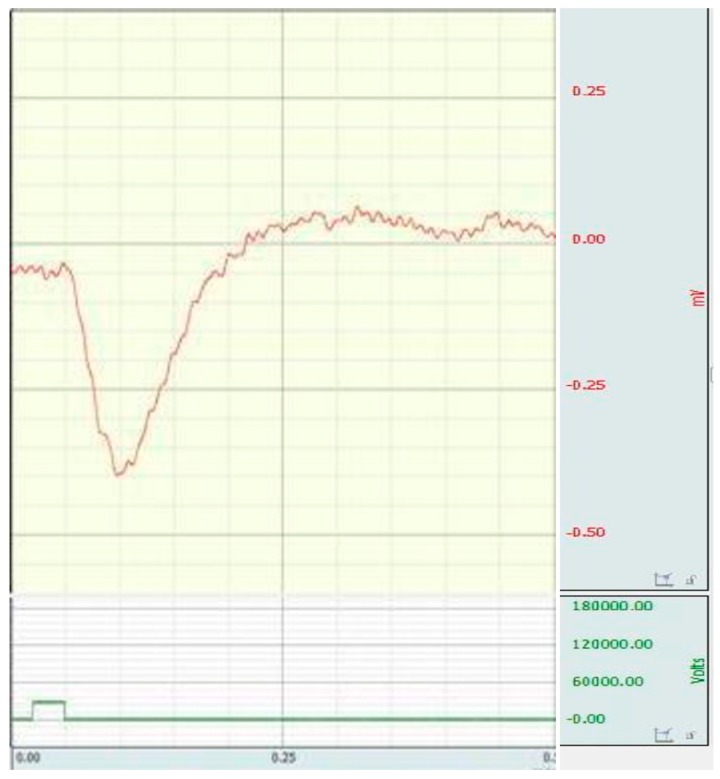
Electroretinogram of rat with the simulated retinal ischemia-reperfusion (inhibition of b-wave is observed).

**Figure 3:**
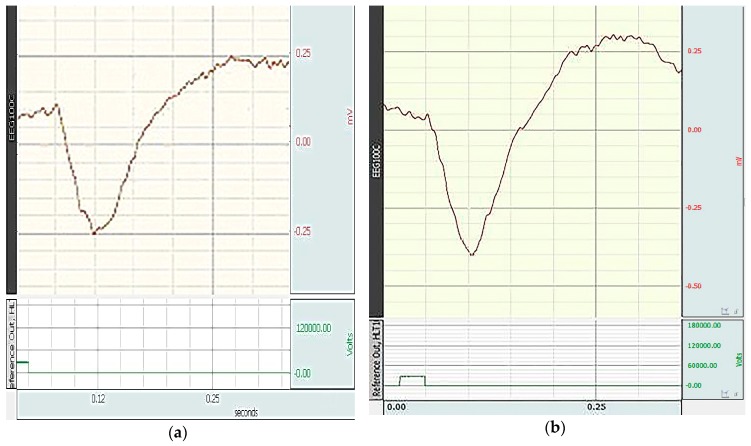
Electroretinogram of rats with the correction of retinal ischemia-reperfusion: (**a**) by l-isomer of ethylmethylhydroxypyridine malate; (**b**) by Emoxipine.

**Figure 4:**
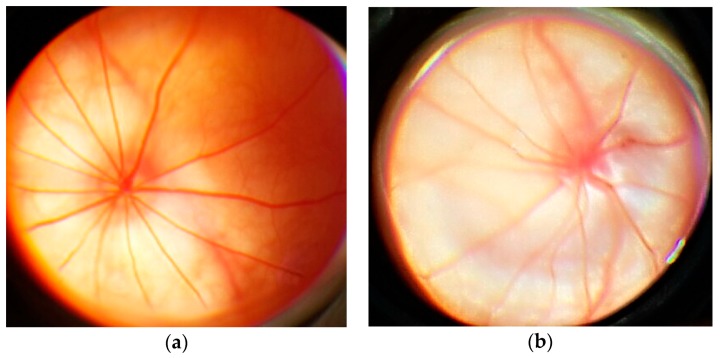
An example of an ophthalmoscopic picture in rats: (**a**) in control group; (**b**) with the simulated retinal ischemia-reperfusion.

**Figure 5:**
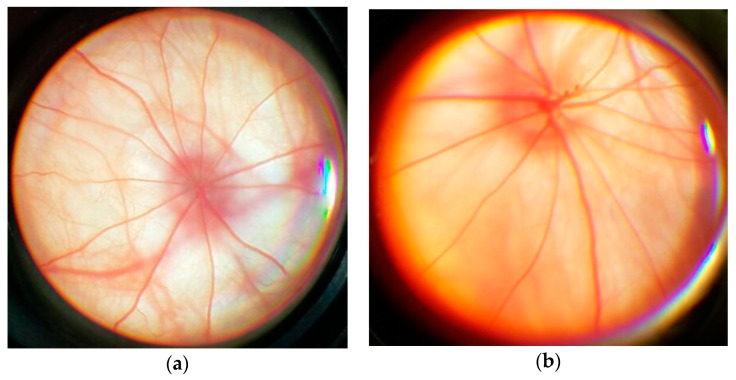
An example of an ophthalmoscopic picture in rats with the simulated retinal ischemia-reperfusion: (**a**) with the correction by l-isomer of ethylmethylhydroxypyridine malate; (**b**) with the correction by Emoxipine.

**Table 1:** Influence of l-isomer of ethylmethylhydroxypyridine malate and Emoxipine on the a and b wave amplitudes when correcting retinal ischemia-reperfusion (M ± m; *n* = 10), mV.

Experimental Groups	The a Wave Amplitudes (*n* = 10)	The b Wave Amplitudes (*n* = 10)
Control	0.35 ± 0.03	0.88 ± 0.08 ^y^
Ischemia-reperfusion model	0.37 ± 0.04	0.44 ± 0.05 *
Ischemia-reperfusion + l-isomer of ethylmethylhydroxypyridine malate, 2 mg/kg	0.35 ± 0.03	0.81 ± 0.07 ^y^
Ischemia-reperfusion + Emoxipine, 2 mg/kg	0.37 ± 0.05	0.78 ± 0.07 ^y^

* *p* < 0.05 compared to the control; ^y^ *p* < 0.05 compared to the ischemia-reperfusion model.

**Table 2:** Influence of l-isomer of ethylmethylhydroxypyridine malate and Emoxipine on the value of the b/a coefficient when correcting retinal ischemia-reperfusion (M ± m; *n* = 10), R.U.

Experimental Groups	Ratio b/a (*n* = 10)
Control	2.5 ± 0.12 ^y^
Ischemia-reperfusion model	1.2 ± 0.04 *
Ischemia-reperfusion + l-isomer of ethylmethylhydroxypyridine malate, 2 mg/kg	2.3 ± 0.16 ^у,#^
Ischemia-reperfusion + Emoxipine, 2 mg/kg	2.1 ± 0.07 ^y^

R.U.: relative units; * *p* < 0.05 compared to the control; ^y^ *p* < 0.05 compared to the ischemia-reperfusion model; # *p* < 0.05 compared to the group with Emoxipine.
